# Association mapping of plant sex and cross-compatibility related traits in white Guinea yam (*Dioscorea rotundata* Poir.) clones

**DOI:** 10.1186/s12870-022-03673-y

**Published:** 2022-06-15

**Authors:** Asrat Asfaw, Jean M. Mondo, Paterne A. Agre, Robert Asiedu, Malachy O. Akoroda

**Affiliations:** 1 International Institute of Tropical Agriculture (IITA), Ibadan, 5320 Nigeria; 2 Institute of Life and Earth Sciences, Pan African University, University of Ibadan, Ibadan, 200284 Nigeria; 3 Department of Crop Production, Université Evangélique en Afrique (UEA), Bukavu, 3323 Democratic Republic of Congo; 4 Department of Agronomy, University of Ibadan, Ibadan, 200284 Nigeria

**Keywords:** Candidate genes, Cross-pollination success, *D. rotundata*, Population structure, Sex determination

## Abstract

**Background:**

White Guinea yam (*Dioscorea rotundata*) is primarily a dioecious species with distinct male and female plants. Its breeding is constrained by sexual reproduction abnormalities, resulting in low success rates in cross-pollination. An accurate method for early detection of this plant’s sex and compatible fertile parents at the seedling stage would improve levels of cross-pollination success in breeding. We used the genome-wide association studies (GWAS) to dissect the molecular basis of plant sex and cross-compatibility-related traits in a panel of 112 parental clones used in *D. rotundata* crossing blocks from 2010 to 2020.

**Results:**

Population structure and phylogeny analyses using 8326 single nucleotide polymorphism (SNP) markers grouped the 112 white yam clones into three subpopulations. Using Multi-locus random-SNP-effect Mixed Linear Model, we identified three, one, and three SNP markers that were significantly associated with the average crossability rate (ACR), the percentage of high crossability (PHC), and the plant sex, respectively. In addition, five genes considered to be directly linked to sexual reproduction or regulating the balance of sex hormones were annotated from chromosomal regions controlling the assessed traits. This study confirmed the female heterogametic sex determination (ZZ/ZW) system proposed for *D. rotundata*.

**Conclusions:**

This study provides valuable insights on the genomic control of sex identity and cross-pollination success in *D. rotundata*. It, therefore, opens an avenue for developing molecular markers for predicting plant sex and cross-pollination success at the early growth stage before field sex expression in this crop.

**Supplementary Information:**

The online version contains supplementary material available at 10.1186/s12870-022-03673-y.

## Background

Creating variability for selection in plant breeding is achieved mainly through the hybridization of selected parents. However, in root and tuber crops, the ability for sexual reproduction (flowering, fertility, synchronization, and compatibility) was substantially affected as a consequence of the domestication process, which favored vegetative propagation at the expense of botanical seeds [1, 2]. During the domestication process, traits related to sexual reproduction were neglected (not maintained), or in some contexts, directly counter-selected due to the associated costs [1–3]. Such reproductive abnormalities are acute in yam (*Dioscorea* spp.), a multispecies tuberous crop with substantial economic and socio-cultural importance in the tropics and subtropics [4].

White Guinea yam (*D. rotundata*) is the most widely grown yam species, accounting for ~ 80% of the total food yam production worldwide [5, 6]. It is characterized by different ploidy levels (2× and 3×) with a basic chromosome number of 20 [7]. Its propagation is through both sexual and asexual means. The sexual reproduction involving the plant’s floral parts is predominantly dioecious (with distinct male and female plants), although monoecious individuals possessing both male and female flowers exist [7–9]. As in other yam species, the ability for sexual reproduction of *D. rotundata* was substantially altered due to the predominantly asexual propagation involving a vegetative part of a plant: tubers and vines. For instance, there are about 58% flowering genotypes in a population randomly sampled from genebank accessions, breeding lines, and landraces [8–10]. Of the genotypes that flower, ~ 60% are males, ~ 29% females, and ~ 11% monoecious [9]. Flowering in *D. rotundata* is characterized by a female heterogametic sex determination system (ZZ/ZW), and maleness is the default phenotype [7]. Hence, monoecy could be expressed as the failure of the W allele to feminize a subset of flowers [7, 11]. The ZW individuals can potentially change sex over time and across locations, indicating that the Z-suppressing function can be affected by the environment. The phenomenon of sex switching in the yam crop across years and locations complicates crossing designs in pipelines of population improvement [12]. Hence, an accurate diagnosis of sex type at the early growth stages in plants is crucial for an efficient crossing plan in *D. rotundata* breeding programs.

Sex types in plants could be identified using phenotypic or molecular markers. Distinguishing or predicting sex types in yam plants using phenotypic markers is less accurate, delayed in expression, and often influenced by growth environments [8, 12]. Molecular markers are, therefore, the best options for early detection of sex in yam breeding [7, 8, 10, 12–14]. Previous attempts at introducing marker-assisted selection for sex detection identified a female-specific marker (sp16) and a male-specific marker (sp1) on the pseudo-chromosome 11, a 17 Mb long chromosome estimated from a diploid female genotype TDr96_F1 reference genome [7]. Based on previous reports, the prediction accuracy of these markers is not always perfect since sex determination in *D. rotundata* is a multi-genic trait [8, 11, 12]. In addition, the phenotypic sex switch across environments is another indication that sex expression in white yam is multi-genic and still under evolution [15]. As suggested by Denadi et al. [12], identifying more sex markers is thus encouraged for accurate identification.

Previous studies on flowering and sex determination in *D. rotundata* used bi-parental populations [7] and thus there is a chance that results could have been related to the parental specificity. In addition to the flower sex expression, the low crossability rate among cultivars which refers to the success rate in terms of fruit or seed set in cross-combinations is a significant challenge in yam genetic improvement efforts through breeding. The overall crossability rate at the International Institute of Tropical Agriculture (IITA), Nigeria, for the white yam crossing block between 2010 and 2020 was estimated at ~ 23% [14, 16]. However, little is known about the genetic basis underlying cross-compatibility in *D. rotundata*. Hence, this study employed genome-wide association studies (GWAS) on a diversity panel of white Guinea yam parental clones used in crossing blocks for 11 years (2010–2020) at IITA to identify chromosomal regions linked to sex identity and cross-pollination success.

## Results

### Phenotypic and genotypic profile of the population

#### Variation of the phenotypic traits in the study panel

**Fig. 1:**
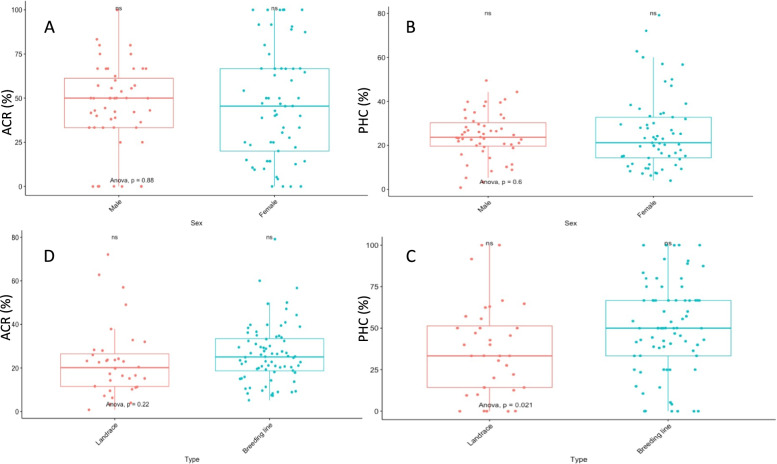
Variations of ACR and PHC with the study panel based on genotypes’ breeding status and sex: **A** ACR variations across plant sexes, **B** PHC variations across plant sexes, **C** PHC variations across breeding statuses, **D** ACR variations across breeding statuses

The average crossability rate (ACR) ranged from 0.8% on the landrace *Ehobia* to 79.2% on the breeding line TDr1689039AB, with a mean of 25.2%. The percentage high crossability (PHC) varied from 0 to 100%, with a mean of 45.9%. The male flowering clone TDr9501932 and the female flowering clone TDr9700917 were the most used parents, having been involved respectively in 51 and 44 cross-combinations. Breeding lines had generally higher crossability indices (mean PHC = 50.1%, ACR = 26.3%) compared with landraces (PHC = 37.1%, ACR = 22.8%). These indices were less influenced by plant sex (Fig. 1, Table S1, Table S2). A positive and significant correlation value (*r* = 0.87, *P* < 2.2e-16) was observed between ACR and PHC.

#### Genetic diversity and population structure of the study panel

A total of 8326 filtered SNPs randomly distributed across the 20 *D. rotundata* chromosomes were used for this study. The highest number of SNPs was identified on chromosome 5, followed by chromosomes 8 and 4 (Fig. S1). The lowest number of SNPs (177) was obtained on chromosome 11. High SNP marker density (red region) was observed across all the 20 chromosomes. An average heterozygosity value of 0.10 was found for genotypes, with the lowest heterozygosity value (0.07) being recorded on breeding line TDr9619158 and the highest (0.16) on breeding line TDr1684005AB. The linkage disequilibrium (LD) analysis showed the presence of 400,765 pairs of loci within a physical distance extending up to 9761.13 bp and 2292 pairs of loci were in complete LD (*R*^2^ = 1). Pearson’s correlation coefficients showed low and positive correlations (*r* = 0.034) between the LD (R^2^) and the physical distance (bp) while *r* = 0.35 was obtained between the R^2^ and the LD, indicating the existence of a linkage decay (Fig. S2). Assessment of the LD plotting showed an average LD value of *R*^2^ = 0.029 and drops to background level (*R*^2^ < 0.03) across the genome.

**Fig. 2:**
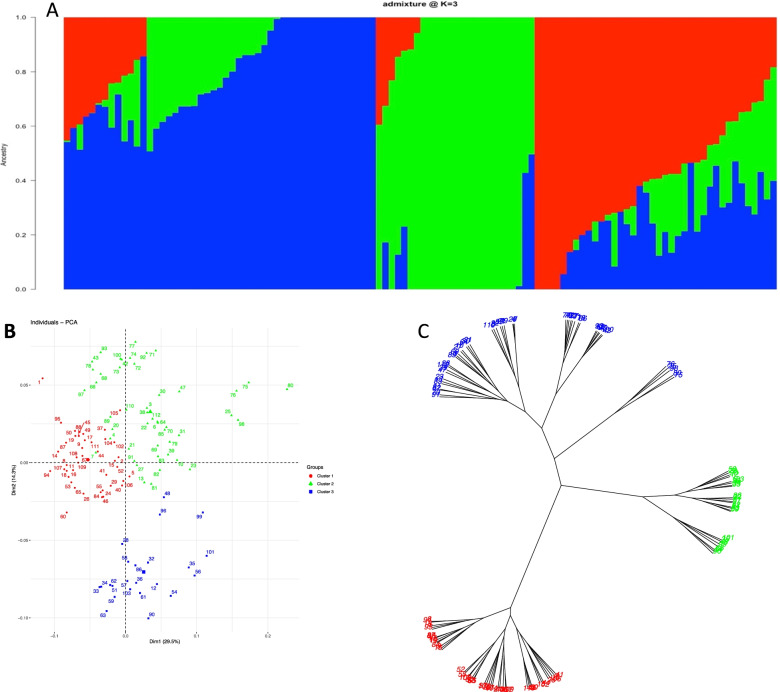
Population structure for 112 *D. rotundata* genotypes based on admixture analysis using 8326 SNPs. **A** population structure based admixture at k = 3; **B** principal component analysis and (**C**) phylogenetic tree clustering-based unrooted method. Each color represents a different cluster and the numbers are codes for clones as in Table S3

Population structure analysis used the approaches of cross-validation, admixture, phylogenetic tree clustering-based unrooted method, and principal component analysis (PCA). All suggested the presence of three clusters as the optimal number of genetic groups within the studied white yam panel (Fig. S3, Fig. 2). Through admixture analysis, only a few genotypes (11 in total) were considered as admixed with an ancestry probability < 0.5 (Fig. 2). The membership probabilities for assigning genotypes into a particular group ranged from 0.50 to 0.99. With 49 genotypes, Cluster 1 had the highest number and consisted of accessions that were genetically distinct from those in clusters 2 and 3.

Clustering analysis using the unrooted phylogenetic tree clustering method discriminated the entire population into three clusters (Table S3). Cluster 1 (42 members) was comprised mainly of landraces; cluster 2 (47 members) was dominated by breeding lines, while cluster 3 (23 members) had only breeding lines.

#### Loci associated with ACR, PHC, and flower sex

**Fig. 3:**
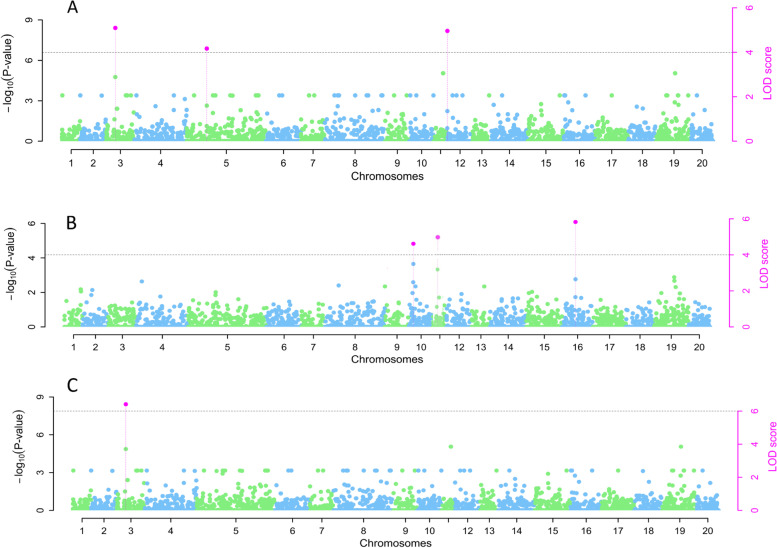
GWAS showing the Manhattan plots associated with (A) ACR, (B) plant sex and (C) PHC. The red dots above the horizontal line indicated the SNP markers associated with the related

The marker–trait association analysis identified three unique SNPs, respectively, on chromosomes 3, 5, and 12 that were significantly associated with the ACR. The phenotypic variance explained (PVE) by these markers ranged from 0.0 to 12.78%. One SNP on chromosome 3 was linked to the PHC, explaining 19.87% of the phenotypic variance. For the plant sex, we identified three SNP markers located on chromosomes 10, 11, and 16 (Fig. 3, Table 1). Of the six different genetic models adopted for the marker-trait association analysis, the three SNP markers for sex identity were identified by five different models (Table 1). The phenotypic variation ranged from 0.0 to 20.33%.

**Table 1:** Marker-trait associations from GWAS scanning for ACR, PHC and plant sex and associated candidate genes in *D. rotundata*

Trait	Method	Markers	Chr	Marker position (bp)	QTN effect	LOD score	log10(P)	R^**2**^ (%)	MAF	GfC1	Orthologs	Putative function
Sex	mrMLM	chr10_867,049	10	867,049	−0.66	4.61	5.40	20.33	0.46	C	*ABA_WDS*	Controls sex before transpiration in vascular plants
	mrMLM	chr16_9,051,658	16	9,051,658	0.92	5.82	6.65	0.00	0.15	C		
	FASTmrMLM	chr10_867,049	10	867,049	−0.54	4.50	5.27	13.81	0.46	C		
	FASTmrMLM	chr16_9,051,658	16	9,051,658	0.71	4.45	5.22	0.00	0.14	C		
	FASTmrEMMA	chr10_867,049	10	867,049	−1.09	4.61	5.39	9.90	0.46	C		
	FASTmrEMMA	chr16_9,051,658	16	9,051,658	1.61	5.82	6.65	13.25	0.14	C		
	pLARmEB	chr10_867,049	10	867,049	−0.55	5.54	6.36	14.29	0.46	C		
	pKWmEB	chr10_867,049	10	867,049	−0.55	5.60	6.42	20.22	0.46	C		
	mrMLM	chr11_4,499,228	11	4,499,228	0.11	4.79	5.99	11.23	0.27	C	*WD40_repeat*	Key regulator of plant-specific developmental events
ACR	FASTmrEMMA	chr03_4,353,133	3	4,353,133	54.81	5.09	5.89	12.78	0.12	C		
	pLARmEB	chr05_15,917,143	5	15,917,143	−7.80	4.16	4.92	2.11	0.11	T	*ATS3*	Plant seed-specific proteins
	pLARmEB	chr12_140,889	12	140,889	10.06	4.95	5.75	0.00	0.11	G	*Cupin_1*	Plant seed storage proteins
											*WRKY_dom*	Involved in the gibberellic acid-induced expression
PHC	FASTmrEMMA	chr03_4,353,133	3	4,353,133	68.35	6.41	7.26	19.87	0.02	C		

*ACR* average crossability rate, *PHC* percentage high crossability, *Chr* chromosome, *R*^*2*^ phenotypic variance explained, *QTN* quantitative trait nucleotide, *MAF* minor allele frequency, *GfC1* genotype for code 1

#### Candidate gene identification

**Fig. 4:**
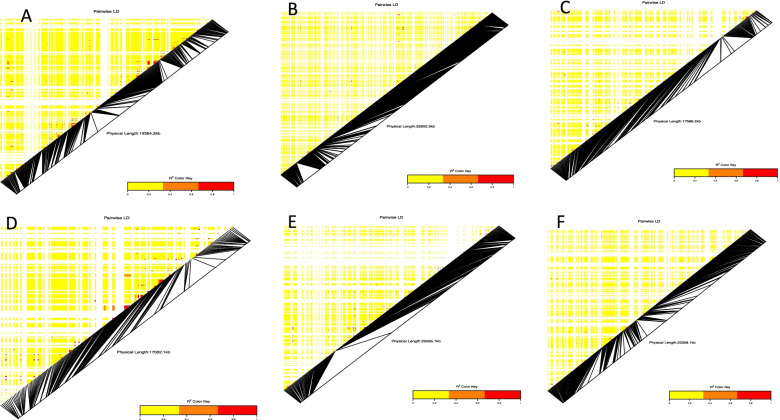
Heatmap LD haplotype blocks for different SNP markers located on different chromosomes associated with ACR, plant sex, and PHC. **A** chromosome 3 was associated with the ACR and the PHC, **B** chromosome 5 associated with the ACR, **C**, **D** & **F** chromosomes 10, 11 and 16 associated with the plant sex, **E** chromosome 12 associated with the ACR. The R^2^ color key indicates the degree of significant association with the putative genes

We identified five candidate genes involved in plant reproduction and hormone regulation within the same linkage block controlling target traits (Fig. 4; Table 1). Of these, *ATS3, Cupin_1,* and *WRKY_dom* were annotated on chromosomes controlling the ACR. Two candidate genes, *ABA_WDS* and *WD40_repeat*, were mapped on chromosomes associated with the plant sex while no candidate gene was located in the region linked to PHC.

#### SNP markers effect prediction

**Fig. 5:**
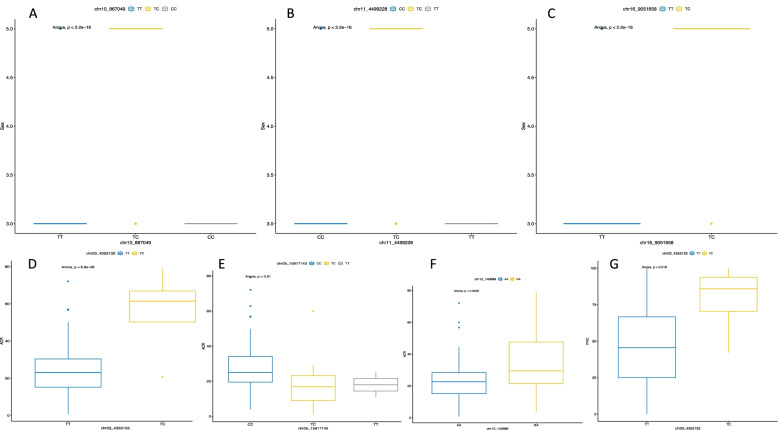
Marker prediction for target traits in *D. rotundata*: **A**–**C** markers associated with plant sex, **D**–**E** markers associated with ACR and (**G**) marker associated with PHC. The boxplots represent the segregation probabilities for each marker. The values above the graphs represent the *P*-values. For analysis purposes, the male sex was coded as 3 and the female sex as 5

Femaleness was associated with heterozygosity for all sex markers, while males were determined by homozygote alleles (Figs. 5A–C). For instance, female clones were from 91.8 to 98.4% heterozygous for markers linked to sex determination (Fig. S4) while these markers displayed 92.2–100% homozygosity in the male genotype population (Fig. S5). Based on these results, sex determination in *D. rotundata* was primarily controlled by the female parents. Of the three candidate markers associated with ACR, the overall tendency showed that high ACR was associated with heterozygosity (Figs. 5D–F). The trend was the same for the one SNP marker associated with the PHC (Fig. 5G).

## Discussion

This study allowed the identification of chromosomal regions and candidate genes underlying sex identity and cross-pollination success in yam. Based on the haplotype segregation analysis of the three markers associated with ACR, high cross-pollination success in white yam was mainly predicted by heterozygote alleles. Moreover, a marker for PHC on chromosome 3 significantly predicted probabilities of high or low cross-pollination success in the *D. rotundata* panel. These markers should, therefore, be targeted for genotypes profiling for cross-compatibility to improve cross-pollination success in *D. rotundata*. These findings agreed with a previous report on *D. alata* which showed the effectiveness of SNP markers to predict both ACR and PHC [14].

Based on the allele variant segregation analyses, sex in *D. rotundata* was controlled by the female parent since more than 90% of female clones were heterozygous for sex markers while the male population was 92–100% homozygous. Using a different method (GWAS) and different plant materials, this study confirmed the female heterogametic sex determination (ZZ/ZW) system proposed for *D. rotundata* yam [7, 11, 17]. In such a system, ZW determines female sex phenotype and ZZ the male sex phenotype. It is noteworthy that Tamiru et al. [7] used QTL-seq analysis on a bi-parental F_1_ progeny segregating for sex, while in this study we performed GWAS using genotyping-by-sequencing (GBS) and a diverse panel of 112 *D. rotundata* cultivars, including landraces and breeding lines. Our study was, therefore, strengthening previous conclusions on the *D. rotundata* sex determination system and dismissed our hypothesis that the outcomes of previous studies could have been affected by parental specificity. Promising sex markers could be converted into low cost Kompetitive Allele-Specific PCR (KASP-PCR) markers, then validated and used for sex phenotype prediction at the early seedling stages of white Guinea yam. Successfully validated markers should then be used in yam breeding to complement the sp16 (associated with the female allele) and sp1 (associated with the maleness) genetic markers previously proposed by Tamiru et al. [7]. As stated in the introduction, early use of previously developed markers did not always result in accurate sex prediction [8, 11, 12]. Among emerging hypotheses to explain their limited efficiency, we had the multi-genic nature of sex determination in *D. rotundata* [12] and the strong influence of environmental factors on flowering pattern and sex identity. This strong environmental influence often leads to instability of the sex or sex-switch across locations and years/generations, a situation that undermines experimental designs in crossing blocks [12]. These hypotheses pressed, therefore, for more markers to be combined in sex prediction. The outcome from the current study provided additional molecular markers on chromosomes 10, 11, and 16 to help improve sex prediction. Like the sp16 marker, all the sex markers from this study provided strong potential in predicting femaleness. One of the markers mapped was on the same chromosome to that of the sp16 as reported by Tamiru et al. [7]. The two complementary markers were identified from chromosomes 10 and 16. Further studies would be necessary for developing markers detecting monoecious sex phenotype despite the strong involvement of the environment on its expression since monoecy is a recurrent phenomenon in white Guinea yam.

However, another hypothesis to be considered on why sex prediction is difficult in yam, in addition to those stated above (multi-genic nature and environmental influences), is the possibility of partial sex-linkage. If sequence variants are not completely sex-linked, they may be quite useful but will not diagnose sex with 100% reliability. If the evidence of sex-linkage in yam is not established well, these problems of difficult sex prediction cannot probably be ameliorated by developing more markers that show associations with sex. More markers will not aid sex prediction if the markers are fully sex-linked — after all, just one such marker will be sufficient. Therefore, the argument for more markers as suggested by Agre et al. [8]; Denadi et al. [12] and Sugihara et al. [11] would need a bit more thought. One speculative possibility is that there could be a major female-determiner (defining a W-linked region), plus a closely linked factor (or several factors) that improve female functions. Then maybe a genotype that has both factors will be a stable female whose sex can perhaps be predicted reliably by markers in the region, while a genotype that has just the first factor will be less stable, and might sometimes have a male phenotype. Also, based on previous reports, there is a shift of sex determining locus across yam species. For example, recent studies on *D. alata* (using the GWAS) mapped significant sex-linked QTNs on chromosome 6 with a XX/XY sex-determination system [2, 18]. These findings agreed with the conclusions from the quantitative trait loci (QTL) approach in two biparental populations [19]. Since the locations of sex determining locus are on a different chromosome in another species, this might suggest either that a gene may move from one chromosome to another (as was inferred in strawberry species [20, 21], another polyploid plant), or that new genes can replace existing sex-determining genes. If the latter is the case, this might be another possible explanation for multi-gene control of sex-determination in *D. rotundata* (the different locations might reflect populations or species that are in transitional states, having gained a new sex determination gene location, but not yet lost the old one). These above-mentionned hypotheses represent a brief outline of some possibilities to test in the future for better understanding of the nature and factors controlling sex in white yam.

Cormier et al. [2] hypothesized that sex chromosome in yam is heteromorphic as putative highly male specific structural variants were detected between the sequenced male pools and the reconstructed male chromosome 6 of *D. alata*. No cytological evidence exists for *D. rotundata* on whether the sex chromosome 11 is acrocentric or metacentric, heteromorphic or homomorphic, due partly to the small size of *Dioscorea* chromosomes. In depth studies are, therefore, needed for a better understanding of the nature and structure of the sex chromosomes in white yam. The gene annotation allowed us to identify candidate gene/protein families associated with sex and cross-pollination in *D. rotundata*. Most of these candidate genes were involved in the regulation of hormones (such as the gibberellins, auxins, ethylene, abscissic acid, and cytokinins) influencing sex identity and sexual reproduction in plants. Indeed, previous experiments have shown that ethylene and auxins increased femaleness in dioecious and monoecious plants while cytokinins and gibberellins have masculinizing effects [15, 22–24]. However, there is need for multi-omics data analysis to validate the candidate genes instead of merely relying on online database and literature. Regarding PHC and ACR as indices for cross-pollination, no quantitative trait loci (QTL) have yet been reported for *D. rotundata*, thus the information provided in the present study would open an avenue in developing genomic tools for predicting these cross-compatibility indices in yam breeding programs. Once these markers are validated, they would support breeding programs in controlling the generally low cross-pollination success reported for *D. rotundata* species. Indeed, Mondo et al. [14, 16] show, while using 11-year crossing data, that cross-pollination success rates for the two major yam species are ~ 23 for *D. rotundata* and 31% for *D. alata*.

## Materials and methods

### Plant materials and phenotypic data collection

In this study, 112 *D. rotundata* genotypes ranging from landraces to advanced breeding lines were used, among which 61 were females and 51 were males. The 112 genotypes (possessing both phenotypic and sequencing information) were selected from a set of 426 clones being used as parents in crossing blocks at the IITA white yam breeding program for the period from 2010 to 2020. More information on the IITA breeding sites is presented in Table S4. For the entire period of data collection, yam crossing blocks were established between April and May and flowering occurred from late July to mid-October. The field management followed the standard recommendations for the yam crop [18]. The land was tilled and ridged with 1 m spacing; ~ 150 g tuber setts were planted on top of the ridge. Individual plants were staked.

**Fig. 6:**
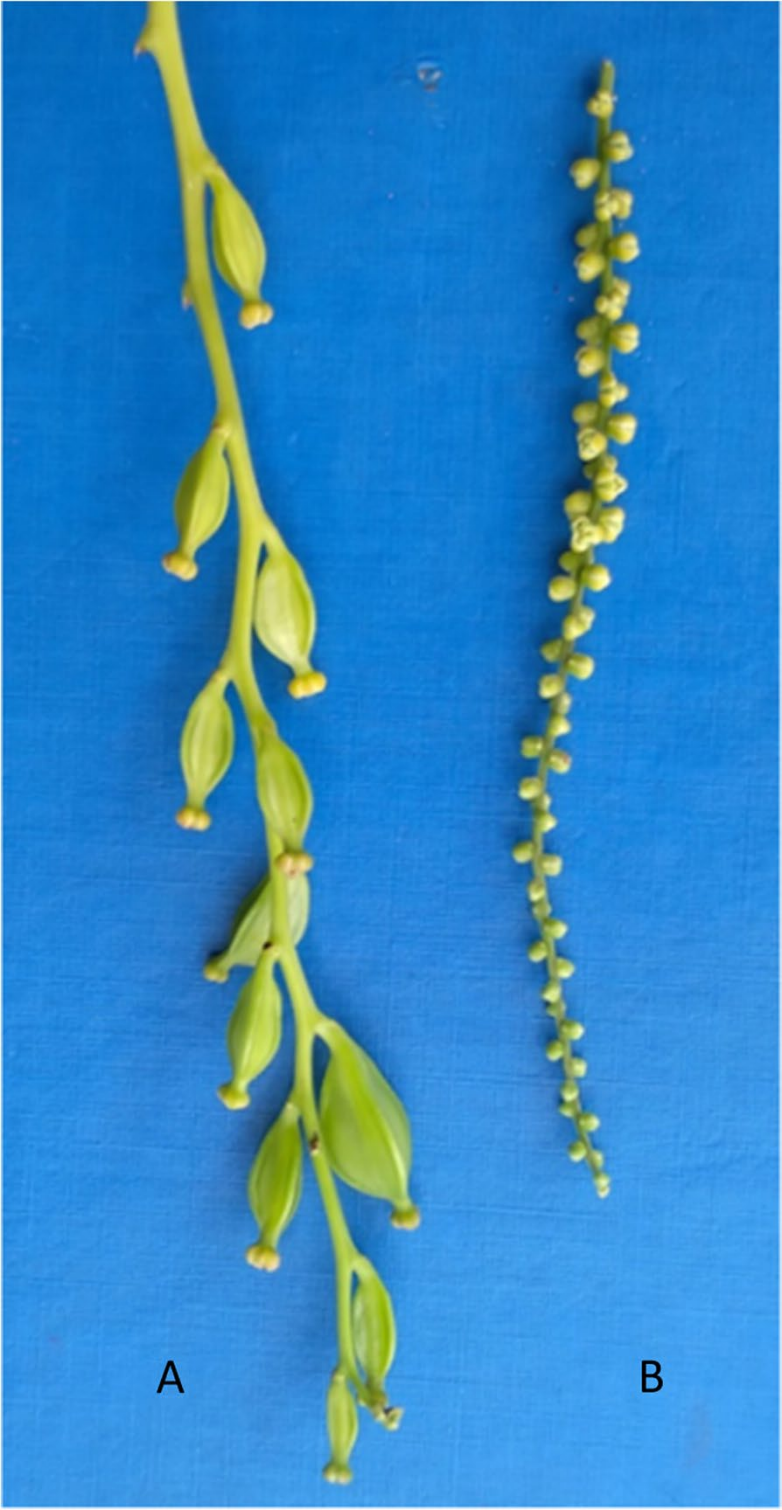
Flower dimorphism in *D. rotundata*: **A** spike with female flowers, **B** spike with male flowers

The plant sex phenotype was scored at flowering by visual observations as directed by the yam crop ontology [25]. It was noteworthy that yam’s male and female flowers were morphologically different (in size and shape), the female flower being larger than the male counterpart (Fig. 6). The sex phenotype was scored as 1 for non-flowering, 2 for male, 3 for female, 4 for monoecious male, and 5 for monoecious female [25]. For convenient analyses, we focused only on genotypes with stable flowering over the considered period, thus excluding the non-flowering and monoecious, and those with irregular/erratic flowering patterns.

The cross-compatibility indices, such as ACR and PHC, were estimated using the 11-year crossing block data from the IITA Yam Breeding Unit. The calculations were performed as in Mondo et al. [14, 16]. The ACR consisted of dividing the sum of means of a genotype’s crossability rates by the number of cross-combinations in which it was involved from 2010 to 2020:

1 $$ACR=\frac{\sum Crossability\;rates}{Number\;of\;cross\;combinations}$$

In eq. (1), the crossability rate was calculated as follows:

2 $$Crossability\;rate\;\left(\%\right)=\frac{Number\;of\;fruits\;set}{Number\;of\;flowers\;pollinated}\times100$$

The crossability in this study refers to the hybridization success with a genotype in cross-combinations in terms of fruit and seed set. It therefore translated as the ability of parental genotypes as pollen source or recipients in cross-combinations result in a fruit set as consequence of the absence of pre- and post-zygotic barriers, earlier reported for yam species and cultivars within species [16].

The PHC for a parental genotype was estimated as the number of times the crossability rate exceeded the species overall cross-compatibility, divided by the number of cross-combinations in which that parental genotype was involved:

3 $$PHC\;\left(\%\right)=\frac{Number\;of\;crossability\;rates>overall\;species'\;mean}{Number\;of\;cross\;combinations}\times100$$

Based on previous reports, the overall crossability rate for *D. rotundata* is 23.4% [14, 16]. The pollination information (ACR and PHC) of genotypes used in this study is presented in Table S2. This information was summarized (by mean ± standard deviation) using a cross-tabulation function implemented in Microsoft Excel.

### Genotyping of the GWAS panel

In this study, we used historical genotypic data from previous genotyping-by-sequencing [26]. In total, 117,078 SNP markers were assembled and subjected to SNP marker filtering. Markers with low sequence depth < 5; missing values > 20%; minor allele frequency (MAF) < 5%, paralog (1.5) and Indel SNP markers were removed. This quality control filtering resulted in 8326 SNPs distributed across the 20 chromosomes which were retained for further analyses.

### Population structure and genetic diversity analysis

Three methods were used to assess the population structure and the genetic diversity among the genotypes. These were the model-based maximum likelihood estimation of ancestral subpopulations through STRUCTURE HARVESTER [27], the phylogeny tree using APE (analyses of phylogenetics and evolution) library package [28] and the PCA using FactorMiner R package [29].

Structure software version 2.3.3 [30, 31] was used to cluster genotypes into subpopulations. Structure simulations were carried out using a burn-in period of 20,000 iterations and a Markov chain Monte Carlo (MCMC) set at 20,000. A binary file was generated using plink and later subjected to cross-validation approaches for population structure analysis. Thus, the most likely *K* value was determined. A cut-off value of 50% (ancestry value) was applied and used to estimate membership probabilities; genotypes were assigned to groups accordingly. Population structure was then plotted using bar plot function implemented in R. For the PCA, the number of clusters was assessed using the “silhouette” function implemented in FactoMiner R package [29].

### GWAS for plant sex, ACR and PHC

All the phenotypic and genotypic information was used to detect the quantitative trait nucleotides (QTNs) using multi-locus models, multi-locus random-SNP-effect MLM (mrMLM), in mrMLM v4.0 (https://cran.r-project.org/web/packages/mrMLM.GUI/index.html) [32, 33]. The GWAS was performed using the R package mrMLM v4.0.2 [33] with six multi-locus models, including: 1) multi-locus random-SNP-effect Mixed Linear Model [32], 2) Fast multi-locus random-SNP-effect EMMA (FASTmrEMMA) [34], 3) Iterative Sure Independence Screening EM-Bayesian LASSO (ISIS EM-BLASSO) [35], 4) polygenic-background-control-based least angle regression plus empirical Bayes (pLARmEB) [36], 5) polygenic-background-control-based Kruskal-Wallis test plus empirical Bayes (pKWmEB) [37], and 6) fast mrMLM (FASTmrMLM) [34].

As recommended by Wang et al. [32], in the mrMLM analysis, we accounted for population structure (Q) generated from Structure analysis and for the Kinship matrix. For each trait, the optimal number Q value included in the GWAS models was determined based on the highest ΔK value. The percentage of variation explained by the associated marker (R^2^) and the effect of the marker was estimated in the mrMLM (v4.0.2) R package (https://cran.r-project.org/web/packages/mrMLM/index.html).

### Candidate gene identification and marker effect prediction

The candidate putative genes associated with SNP markers for target traits were searched within a window range of 1 Mb (upstream and downstream) from *D. rotundata* generic feature format (GFF3) of the reference genome v2 [17] using the SNPReff. LD heat map package [38] was used to perform LD and produced a graphical display, as a heatmap, of pairwise LD measurements among SNPs with significant association for each of the traits independently. Functions of the genes associated with the identified SNPs were determined using the public database Interpro, European Molecular Biology Laboratory-European Bioinformatics Institute (EMBL-EBI). The pairwise LD estimates across chromosomes for significantly associated markers were investigated and plotting was done based on base pairs (bp) distance, using “ggplot2” package in R [39].

Allele variants associated with significant QTL were developed using the “rstatix” package implemented in R. The variant effect prediction was evaluated through the adjusted posterior probability and visualized using ggplot2 R packages.

## Supplementary Information

**Additional file 1.** 

## Data Availability

The Variant Call Format (VCF) file used for analyses can be viewed on www.yambase.org under genotypic data. Phenotypic data associated with the GWAS study are presented as a supplementary file within the document.

## Abbreviations

SNP: Single nucleotide polymorphism
ACR: Average crossability rate
PHC: Percentage of high crossability
IITA: International Institute of Tropical Agriculture
MLM: Mixed liner model
GWAS: Genome-wide association study
PCA: Principal component analysis
PVE: Phenotypic variation explained
KASP: Kompetitive Allele-Specific PCR
LD: Linkage disequilibrium
MAF: Minor allele frequency
QTL: Quantitative trait locus
GBS: Genotyping-by-sequencing
