# Supplementary material for: Association mapping of plant sex and cross-compatibility related traits in white Guinea yam (Dioscorea rotundata Poir.) clones
Source: BMC Plant Biol. 2022 Jun 15;22:294. doi: 10.1186/s12870-022-03673-y (PMC9199169; doi:10.1186/s12870-022-03673-y)
Supplement: Supplementary file 1 — Additional file 1. [file 12870_2022_3673_MOESM1_ESM.docx]

**Supplementary files**

**Table S1.** ACR and PHC variations based on sex and breeding status

**Table S2.** Type, sex and cross-compatibility indices of *D. rotundata* genotypes used for GWAS

**Table S3.** Members of subpopulations from the *D. rotundata* panel used in GWAS

**Table S4.** Soil and weather characteristics of the IITA yam breeding sites, Nigeria, (2010–2020)

**Fig. S1.** Distribution of SNPs across *D. rotundata* chromosomes

**Fig. S2.** Linkage disequilibrium of total SNP markers across the 20 chromosomes

**Fig. S3.** Cross-validation values considering 1 to 20 delta k values

**Fig. S4.** Haplotype view of markers associated with plant sex in female plant of *D. rotundata*

**Fig. S5.** Haplotype view of markers associated with plant sex in male plant of *D. rotundata*

**Table S1.** ACR and PHC variations based on sex and breeding status

| Parameters | PHC (%) | | | |  | ACR (%) | | | |  |
| --- | --- | --- | --- | --- | --- | --- | --- | --- | --- | --- |
|  | Male | Female | Breeding | Landrace |  | Male | Female | Breeding | Landrace | |
| Min | 0.0 | 0.0 | 0.0 | 0.0 |  | 0.8 | 4.0 | 5.2 | 0.8 | |
| Max | 100.0 | 100.0 | 100.0 | 100.0 |  | 49.5 | 79.2 | 79.2 | 72.1 | |
| Mean | 45.5 | 46.3 | 50.1 | 37.1 |  | 24.4 | 25.8 | 26.3 | 22.8 | |
| SD | 24.0 | 31.3 | 27.5 | 27.5 |  | 10.6 | 16.9 | 13.4 | 16.2 | |
| Median | 50.0 | 45.5 | 50.0 | 33.3 |  | 23.2 | 21.2 | 25.1 | 20.1 | |
| Number | 51 | 61 | 76 | 36 |  | 51 | 61 | 76 | 36 | |

Min: Minimum, Max: Maximum, SD: Standard deviation, PHC: Percentage high crossability, ACR: Average crossability rate.

**Table S2.** Type, sex and cross-compatibility indices of *D. rotundata* genotypes used for GWAS

| **Genotype name** | **Type** | **Sex** | **ACR (%)** | **Cross-combinations** | **PHC (%)** |
| --- | --- | --- | --- | --- | --- |
| Agbanwobe | Landrace | Male | 37.9±28.8 | 24 | 62.5 |
| Akunchi | Landrace | Female | 23.7±16.2 | 27 | 63.0 |
| Aloshie | Landrace | Female | 32.8±25.5 | 11 | 45.5 |
| Alumaco | Landrace | Male | 28.3±15.7 | 14 | 57.1 |
| Ami | Landrace | Female | 49.1±41.9 | 3 | 66.7 |
| AnyamayowaGboko | Landrace | Female | 19.8±16.3 | 7 | 42.9 |
| Awanoba | Landrace | Female | 72.1±13.2 | 3 | 100 |
| AwonbaAuchi | Landrace | Female | 57.0±8.7 | 3 | 100 |
| Cheyol | Landrace | Female | 23.0±9.6 | 3 | 33.3 |
| Ehobia | Landrace | Male | 0.8±1.8 | 10 | 0.0 |
| Ehuru | Landrace | Male | 10.2±13.1 | 3 | 33.3 |
| Fakesta | Landrace | Male | 23.2±19.5 | 26 | 50.0 |
| FakestaNRCRI | Landrace | Male | 11.3±6.0 | 5 | 0.0 |
| Hembakwase | Landrace | Female | 16.4±13.1 | 23 | 30.4 |
| HembakwaseAgyaragu | Landrace | Female | 15.2±16.8 | 7 | 14.3 |
| HembakwaseGboko | Landrace | Male | 22.6±35.3 | 3 | 33.3 |
| Kwashie | Landrace | Female | 20.4±23.6 | 6 | 50.0 |
| Lagos | Landrace | Female | 19.9±10.2 | 5 | 40.0 |
| Meccakusa | Landrace | Female | 9.5±11.3 | 35 | 14.3 |
| Nduu | Landrace | Female | 15.2±13.6 | 29 | 27.6 |
| OjuIyawo | Landrace | Female | 6.3±6.1 | 9 | 0.0 |
| OmiEfun | Landrace | Female | 4.0±4.4 | 6 | 0.0 |
| Pampas | Landrace | Female | 11.6±9.0 | 21 | 9.5 |
| Pepa | Landrace | Female | 32.0±21.1 | 17 | 64.7 |
| Pouna | Landrace | Male | 3.3±4.2 | 10 | 0.0 |
| Punch | Breeding line | Female | 56.7±13.1 | 6 | 100 |
| TDr0000362 | Breeding line | Male | 19.3±18.6 | 18 | 38.9 |
| TDr0500046 | Breeding line | Female | 11.3±16.3 | 4 | 25.0 |
| TDr0500589 | Breeding line | Male | 19.9±9.7 | 3 | 66.7 |
| TDr064 | Landrace | Female | 14.3±11.2 | 7 | 14.3 |
| TDr0700756 | Breeding line | Female | 30.1±10.7 | 3 | 66.7 |
| TDr0800091 | Breeding line | Male | 26.8±19.2 | 4 | 75 |
| TDr0900002 | Breeding line | Male | 14.3±9.9 | 4 | 25 |
| TDr0900052 | Breeding line | Male | 24.9±13.7 | 3 | 33.3 |
| TDr0900070 | Breeding line | Male | 32.4±28.4 | 3 | 66.7 |
| TDr0900134 | Breeding line | Male | 35.0±29.6 | 3 | 66.7 |
| TDr0900324 | Breeding line | Male | 39.8±28.7 | 4 | 50.0 |
| TDr0909132 | Breeding line | Male | 15.9±13.9 | 4 | 50.0 |
| TDr1100497 | Breeding line | Male | 25.2±18.2 | 6 | 50.0 |
| TDr1100873 | Breeding line | Female | 27.8±12.5 | 8 | 75.0 |
| TDr1301550 | Breeding line | Female | 29.3±22.8 | 6 | 50.0 |
| TDr1400359 | Breeding line | Female | 19.6±12.0 | 18 | 38.9 |
| TDr1401220 | Breeding line | Female | 50.0±22.7 | 21 | 90.5 |
| TDr1500031 | Breeding line | Male | 29.6±28.9 | 6 | 50.0 |
| TDr1500042 | Breeding line | Male | 40.9±30.6 | 7 | 57.1 |
| TDr1500043 | Breeding line | Male | 39.5±25.3 | 10 | 80.0 |
| TDr1500096 | Breeding line | Male | 36.2±22.6 | 6 | 83.3 |
| TDr1500100 | Breeding line | Male | 31.0±28.0 | 9 | 44.4 |
| TDr1500101 | Breeding line | Male | 28.8±25.4 | 5 | 60.0 |
| TDr1500128 | Breeding line | Male | 26.4±30.4 | 6 | 50.0 |
| TDr1612105 | Breeding line | Female | 26.9±14.5 | 5 | 60.0 |
| TDr1613401 | Breeding line | Male | 24.7±11.8 | 3 | 66.7 |
| TDr1613701 | Breeding line | Male | 23.5±7.9 | 3 | 33.3 |
| TDr1614001 | Breeding line | Female | 25.2±16.4 | 6 | 66.7 |
| TDr1614205 | Breeding line | Male | 34.0±12.8 | 4 | 75.0 |
| TDr1615003 | Breeding line | Female | 33.3±7.3 | 12 | 91.7 |
| TDr1615116 | Breeding line | Female | 21.2±19.1 | 4 | 25.0 |
| TDr1615502 | Breeding line | Female | 17.9±12.6 | 5 | 40.0 |
| TDr1620004 | Breeding line | Female | 9.3±4.0 | 4 | 0.0 |
| TDr1620006 | Breeding line | Male | 39.8±21.8 | 5 | 80.0 |
| TDr1620009 | Breeding line | Female | 9.6±9.8 | 7 | 14.3 |
| TDr1620015 | Breeding line | Female | 34.3±12.8 | 3 | 66.7 |
| TDr1620029 | Breeding line | Female | 15.0±14.8 | 4 | 25.0 |
| TDr1621001 | Breeding line | Male | 8.3±5.0 | 6 | 0.0 |
| TDr1621012 | Breeding line | Male | 26.1±20.6 | 4 | 50.0 |
| TDr1621019 | Breeding line | Male | 32.4±19.7 | 6 | 66.7 |
| TDr1631C | Landrace | Female | 11.0±9.6 | 8 | 12.5 |
| TDr1669009 | Breeding line | Male | 21.9±16.0 | 12 | 41.7 |
| TDr1669010 | Breeding line | Female | 25.4±16.5 | 6 | 66.7 |
| TDr1679004 | Breeding line | Female | 13.7±10.8 | 3 | 33.3 |
| TDr1680013AB | Breeding line | Male | 18.8±5.8 | 4 | 25.0 |
| TDr1680035AB | Breeding line | Male | 5.2±9.2 | 4 | 0.0 |
| TDr1680036AB | Breeding line | Female | 18.2±16.3 | 6 | 50.0 |
| TDr1683039AB | Breeding line | Male | 21.1±17.1 | 4 | 50.0 |
| TDr1684005AB | Breeding line | Female | 9.0±7.4 | 3 | 0.0 |
| TDr1689009AB | Breeding line | Female | 36.6±17.9 | 6 | 66.7 |
| TDr1689010AB | Breeding line | Male | 10.8±8.1 | 3 | 0.0 |
| TDr1689011AB | Breeding line | Male | 44.3±29.4 | 3 | 66.7 |
| TDr1689021AB | Breeding line | Female | 60.0±23.9 | 5 | 80.0 |
| TDr1689039AB | Breeding line | Female | 79.2±4.8 | 3 | 100 |
| TDr1709A | Landrace | Female | 7.2±10.0 | 10 | 10.0 |
| TDr1717 | Landrace | Male | 17.3±21.0 | 3 | 33.3 |
| TDr2050 | Landrace | Female | 23.3±23.7 | 6 | 50.0 |
| TDr2826A | Landrace | Female | 28.0±23.4 | 17 | 47.1 |
| TDr3010 | Landrace | Female | 62.8±23.4 | 12 | 91.7 |
| TDr8902157 | Breeding line | Male | 8.9±6.8 | 9 | 0.0 |
| TDr8902475 | Breeding line | Female | 7.5±6.8 | 19 | 5.3 |
| TDr8902665 | Breeding line | Female | 7.3±6.8 | 24 | 4.2 |
| TDr8902677 | Breeding line | Male | 20.7±15.3 | 19 | 42.1 |
| TDr9501932 | Breeding line | Male | 22.7±16.8 | 51 | 43.1 |
| TDr9518544 | Breeding line | Female | 22.8±17.7 | 27 | 40.7 |
| TDr9518988 | Breeding line | Female | 10.5±9.8 | 17 | 23.5 |
| TDr9519156 | Breeding line | Female | 20.9±15.2 | 15 | 46.7 |
| TDr9519158 | Breeding line | Female | 47.1±24.6 | 9 | 88.9 |
| TDr9519177 | Breeding line | Female | 39.0±18.0 | 8 | 87.5 |
| TDr9601818 | Breeding line | Male | 22.9±19.1 | 7 | 42.9 |
| TDr9619158 | Breeding line | Female | 38.4±18.9 | 3 | 100 |
| TDr9700205 | Breeding line | Female | 15.2±14.1 | 20 | 15.0 |
| TDr9700588 | Breeding line | Female | 34.7±17.7 | 4 | 100 |
| TDr9700632 | Breeding line | Female | 29.5±23.3 | 24 | 54.2 |
| TDr9700777 | Breeding line | Male | 20.8±14.7 | 21 | 38.1 |
| TDr9700793 | Breeding line | Female | 8.3±8.4 | 28 | 10.7 |
| TDr9700917 | Breeding line | Female | 20.3±17.4 | 44 | 45.5 |
| TDr9700940 | Breeding line | Male | 49.5±18.0 | 5 | 100 |
| TDr9902562 | Breeding line | Male | 26.6±21.9 | 13 | 53.8 |
| TDr9902607 | Breeding line | Male | 23.1±15.5 | 33 | 42.4 |
| TDr9902626 | Breeding line | Male | 27.6±19.3 | 18 | 55.6 |
| TDr9902789 | Breeding line | Male | 20.4±18.5 | 22 | 36.4 |
| TDr9915 | Landrace | Male | 26.0±21.2 | 5 | 40.0 |
| Ufenyi | Landrace | Female | 16.6±13.0 | 18 | 22.2 |
| Yangbedu | Landrace | Male | 23.7±14.8 | 9 | 55.6 |
| YangbeduGunu | Landrace | Female | 24.2±35.9 | 5 | 20.0 |

*ACR=average crossability rate, PHC=percentage high crossability*

**Table S3.** Members of subpopulations from the *D. rotundata* panel used in GWAS

| **Clones** | **CV1** | **CV2** | **CV3** | **cluster** |
| --- | --- | --- | --- | --- |
| TDr1500096 | 0.54114 | 0.006018 | 0.452842 | 1 |
| Kwashie | 0.592884 | 0.00001 | 0.407106 | 1 |
| TDr1500031 | 0.513207 | 0.093409 | 0.393385 | 1 |
| TDr8902475 | 0.634725 | 0.00001 | 0.365265 | 1 |
| TDr8902665 | 0.647623 | 0.000441 | 0.351936 | 1 |
| Meccakusa | 0.678623 | 0.005854 | 0.315523 | 1 |
| Hembakwase | 0.671205 | 0.025199 | 0.303596 | 1 |
| Cheyol | 0.594055 | 0.163527 | 0.242418 | 1 |
| Ufenyi | 0.716303 | 0.043431 | 0.240266 | 1 |
| Hembakwase-Gboko | 0.542714 | 0.243595 | 0.213691 | 1 |
| TDr1679004 | 0.620949 | 0.17266 | 0.206391 | 1 |
| Lagos | 0.524477 | 0.320081 | 0.155442 | 1 |
| TDr1680013AB | 0.856153 | 0.00001 | 0.143837 | 1 |
| TDr1620006 | 0.506982 | 0.493008 | 0.00001 | 1 |
| TDr1614205 | 0.59019 | 0.4098 | 0.00001 | 1 |
| TDr1621012 | 0.614864 | 0.385126 | 0.00001 | 1 |
| TDr1613401 | 0.636712 | 0.363278 | 0.00001 | 1 |
| TDr1613701 | 0.648545 | 0.351445 | 0.00001 | 1 |
| Ehuru | 0.671501 | 0.328489 | 0.00001 | 1 |
| TDr9902789 | 0.672369 | 0.327621 | 0.00001 | 1 |
| TDr9902607 | 0.673702 | 0.326288 | 0.00001 | 1 |
| TDr9902626 | 0.716673 | 0.283317 | 0.00001 | 1 |
| Awonba-Auchi | 0.720909 | 0.279081 | 0.00001 | 1 |
| TDr1631C | 0.731122 | 0.268868 | 0.00001 | 1 |
| TDr9519158 | 0.741037 | 0.258953 | 0.00001 | 1 |
| TDr0900324 | 0.778823 | 0.221167 | 0.00001 | 1 |
| TDr9700940 | 0.800203 | 0.199787 | 0.00001 | 1 |
| Yangbedu | 0.849826 | 0.150164 | 0.00001 | 1 |
| TDr0909132 | 0.861177 | 0.138813 | 0.00001 | 1 |
| Yangbedu-Gunu | 0.861413 | 0.138577 | 0.00001 | 1 |
| TDr99-15 | 0.868874 | 0.131116 | 0.00001 | 1 |
| TDr1500042 | 0.898666 | 0.101324 | 0.00001 | 1 |
| TDr1500101 | 0.95176 | 0.04823 | 0.00001 | 1 |
| TDr1500128 | 0.993361 | 0.006629 | 0.00001 | 1 |
| Agbanwobe | 0.99998 | 0.00001 | 0.00001 | 1 |
| TDr1400359 | 0.99998 | 0.00001 | 0.00001 | 1 |
| TDr1401220 | 0.99998 | 0.00001 | 0.00001 | 1 |
| TDr1621019 | 0.99998 | 0.00001 | 0.00001 | 1 |
| TDr1669009 | 0.99998 | 0.00001 | 0.00001 | 1 |
| TDr1680035AB | 0.99998 | 0.00001 | 0.00001 | 1 |
| TDr1680036AB | 0.99998 | 0.00001 | 0.00001 | 1 |
| TDr1683039AB | 0.99998 | 0.00001 | 0.00001 | 1 |
| TDr1689010AB | 0.99998 | 0.00001 | 0.00001 | 1 |
| TDr1689011AB | 0.99998 | 0.00001 | 0.00001 | 1 |
| TDr9518988 | 0.99998 | 0.00001 | 0.00001 | 1 |
| TDr9519156 | 0.99998 | 0.00001 | 0.00001 | 1 |
| TDr9519177 | 0.99998 | 0.00001 | 0.00001 | 1 |
| TDr9619158 | 0.99998 | 0.00001 | 0.00001 | 1 |
| TDr9700632 | 0.99998 | 0.00001 | 0.00001 | 1 |
| TDr9700588 | 0.00001 | 0.605755 | 0.394235 | 2 |
| TDr1500100 | 0.171495 | 0.503333 | 0.325172 | 2 |
| TDr9700777 | 0.00001 | 0.768173 | 0.231817 | 2 |
| TDr0500046 | 0.12646 | 0.728234 | 0.145306 | 2 |
| TDr9601818 | 0.230328 | 0.646603 | 0.123069 | 2 |
| TDr1615502 | 0.00001 | 0.881896 | 0.118094 | 2 |
| Fakesta | 0.00001 | 0.942888 | 0.057102 | 2 |
| TDr0800091 | 0.00001 | 0.99998 | 0.00001 | 2 |
| TDr0900002 | 0.00001 | 0.99998 | 0.00001 | 2 |
| TDr0900070 | 0.00001 | 0.99998 | 0.00001 | 2 |
| TDr0900134 | 0.00001 | 0.99998 | 0.00001 | 2 |
| TDr1612105 | 0.00001 | 0.99998 | 0.00001 | 2 |
| TDr1614001 | 0.00001 | 0.99998 | 0.00001 | 2 |
| TDr1615003 | 0.00001 | 0.99998 | 0.00001 | 2 |
| TDr1615116 | 0.00001 | 0.99998 | 0.00001 | 2 |
| TDr1620004 | 0.00001 | 0.99998 | 0.00001 | 2 |
| TDr1620009 | 0.00001 | 0.99998 | 0.00001 | 2 |
| TDr1620029 | 0.00001 | 0.99998 | 0.00001 | 2 |
| TDr8902157 | 0.00001 | 0.99998 | 0.00001 | 2 |
| TDr9501932 | 0.00001 | 0.99998 | 0.00001 | 2 |
| TDr9700917 | 0.00001 | 0.99998 | 0.00001 | 2 |
| TDr0900052 | 0.000011 | 0.999979 | 0.00001 | 2 |
| TDr1620015 | 0.010766 | 0.989224 | 0.00001 | 2 |
| TDr1500043 | 0.428644 | 0.571346 | 0.00001 | 2 |
| TDr0500589 | 0.496382 | 0.503608 | 0.00001 | 2 |
| TDr1684005AB | 0.00001 | 0.00001 | 0.99998 | 3 |
| TDr1689021AB | 0.00001 | 0.00001 | 0.99998 | 3 |
| TDr1689039AB | 0.00001 | 0.00001 | 0.99998 | 3 |
| TDr3010 | 0.00001 | 0.00001 | 0.99998 | 3 |
| TDr1669010 | 0.055414 | 0.00001 | 0.944576 | 3 |
| Aloshie | 0.135957 | 0.00001 | 0.864033 | 3 |
| TDr0700756 | 0.143976 | 0.038326 | 0.817698 | 3 |
| Anyamayowa-Gboko | 0.199264 | 0.00001 | 0.800726 | 3 |
| Alumaco | 0.215012 | 0.00001 | 0.784978 | 3 |
| Oju-Iyawo | 0.176255 | 0.071932 | 0.751813 | 3 |
| Nduu | 0.249236 | 0.00001 | 0.750754 | 3 |
| Pouna | 0.25269 | 0.020441 | 0.726869 | 3 |
| Ehobia | 0.082801 | 0.197703 | 0.719496 | 3 |
| Awanoba | 0.284997 | 0.00001 | 0.714993 | 3 |
| TDr1689009AB | 0.238847 | 0.055679 | 0.705474 | 3 |
| TDr0000362 | 0.198405 | 0.107152 | 0.694443 | 3 |
| TDr8902677 | 0.380181 | 0.00001 | 0.619809 | 3 |
| TDr1621001 | 0.355188 | 0.041056 | 0.603756 | 3 |
| Hembakwase-Agyaragu | 0.241743 | 0.163744 | 0.594514 | 3 |
| Pampas | 0.122072 | 0.284777 | 0.59315 | 3 |
| TDr1717 | 0.210695 | 0.209571 | 0.579734 | 3 |
| Fakesta-NRCRI | 0.134107 | 0.289907 | 0.575987 | 3 |
| TDr9700205 | 0.195604 | 0.228426 | 0.57597 | 3 |
| TDr1709A | 0.212276 | 0.249971 | 0.537753 | 3 |
| TDr06-4 | 0.465169 | 0.00001 | 0.534821 | 3 |
| TDr2050 | 0.2114 | 0.254929 | 0.533671 | 3 |
| TDr9518544 | 0.272196 | 0.203891 | 0.523913 | 3 |
| Akunchi | 0.377873 | 0.152154 | 0.469973 | Admixt |
| Omi-efun | 0.453645 | 0.079322 | 0.467033 | Admixt |
| Ami | 0.30071 | 0.2644 | 0.43489 | Admixt |
| TDr2826A | 0.317678 | 0.298626 | 0.383696 | Admixt |
| TDr9700793 | 0.470092 | 0.147317 | 0.382591 | Admixt |
| TDr1100497 | 0.389628 | 0.261391 | 0.348981 | Admixt |
| TDr9902562 | 0.305402 | 0.348111 | 0.346488 | Admixt |
| Pepa | 0.274515 | 0.411965 | 0.31352 | Admixt |
| TDr1301550 | 0.432362 | 0.259767 | 0.307872 | Admixt |
| TDr1100873 | 0.330494 | 0.440154 | 0.229352 | Admixt |
| Punch | 0.397999 | 0.418823 | 0.183178 | Admixt |

**Table S4.** Soil and weather characteristics of the IITA yam breeding sites, Nigeria (2010–2020)

| Parameters | Abuja | Ibadan |
| --- | --- | --- |
| Latitude | 9°10′ N | 7°29′ N |
| Longitude | 7°21′ E | 3°54′ E |
| Soil type | Sandy clay loam | Sandy loam |
| Soil pH | 6.4 | 6.2 |
| Average annual temperature, °C | 26.0 | 25.9 |
| Minimum annual temperature, °C | 21.3 | 22.4 |
| Maximum annual temperature, °C | 32.9 | 31.6 |
| Annual rainfall, mm | 1363.9 | 1546.9 |
| Rainy days per year | 136.5 | 115.6 |
| Annual evaporation, mm | - | 1261.5 |
| Minimum relative humidity, % | 37.0 | 50.2 |
| Maximum relative humidity, % | 78.2 | 92.8 |
| Sunshine, h | - | 5.8 |
| Solar radiation, | 16.6 | 15.5 |
| Wind speed, m s^-1^ | 1.7 | 3.1 |
| Altitude, masl | ~300 | ~220 |


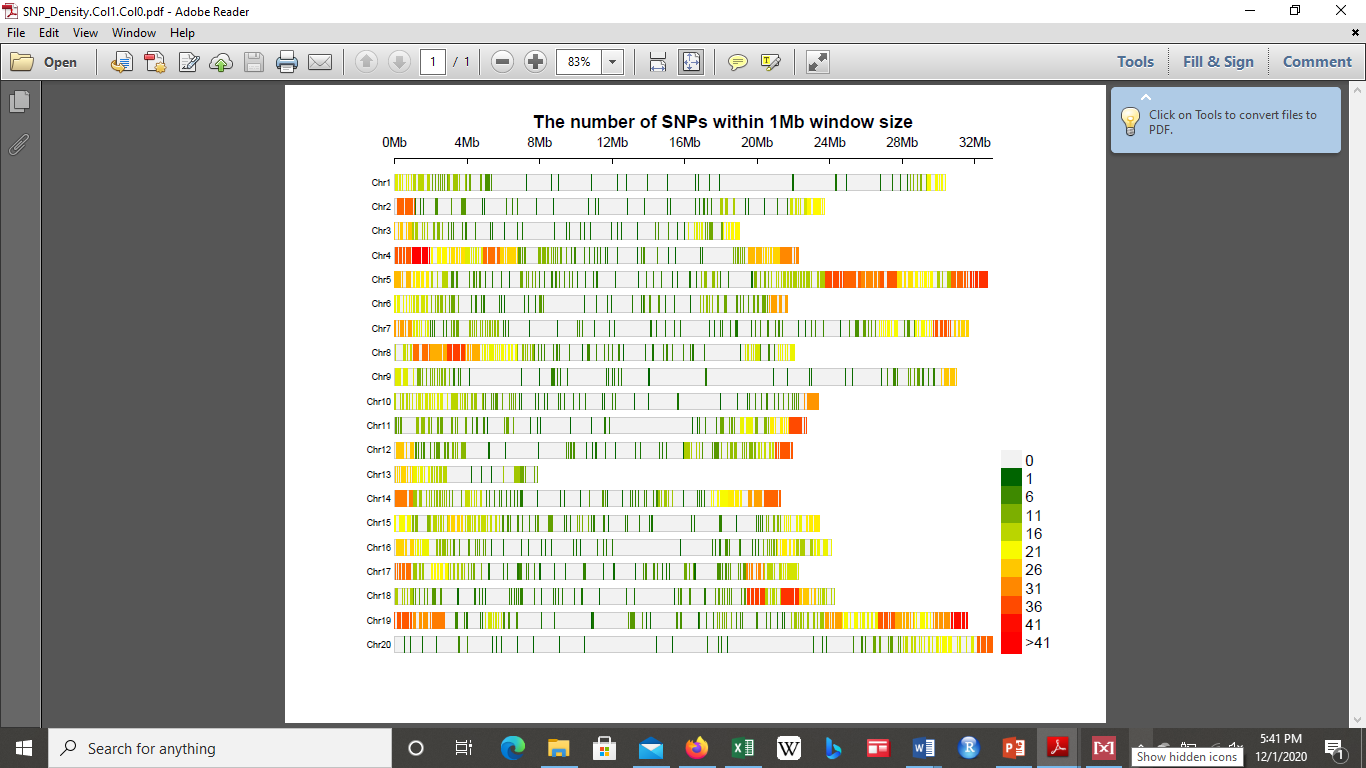


**Fig. S1.** Distribution of SNPs across *D. rotundata* chromosomes

**Fig. S2.** Linkage disequilibrium of total SNP markers across the 20 chromosomes. Trend line of the nonlinear regression of the linkage disequilibrium measure r^2^ versus physical distance (Mb) between single-nucleotide polymorphism (SNP) marker pairs across the 20 yam chromosomes. The LD is computed chromosome-wide.

**Fig. S3.** Cross-validation values considering 1 to 20 delta k values

**
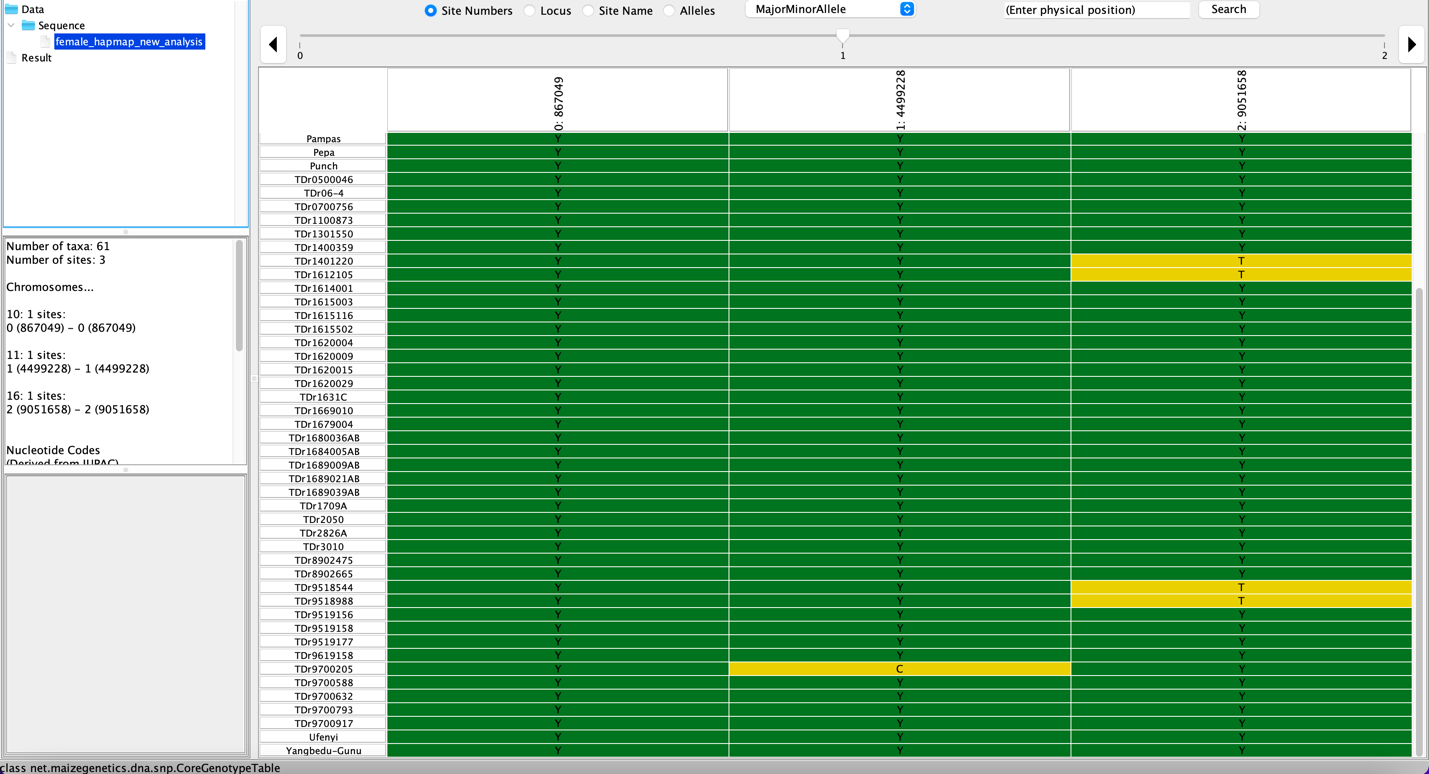
**

**Fig. S4.** Haplotype view of markers associated with plant sex in female plant of *D. rotundata* (green are heterozygote alleles while yellow are for homozygote).


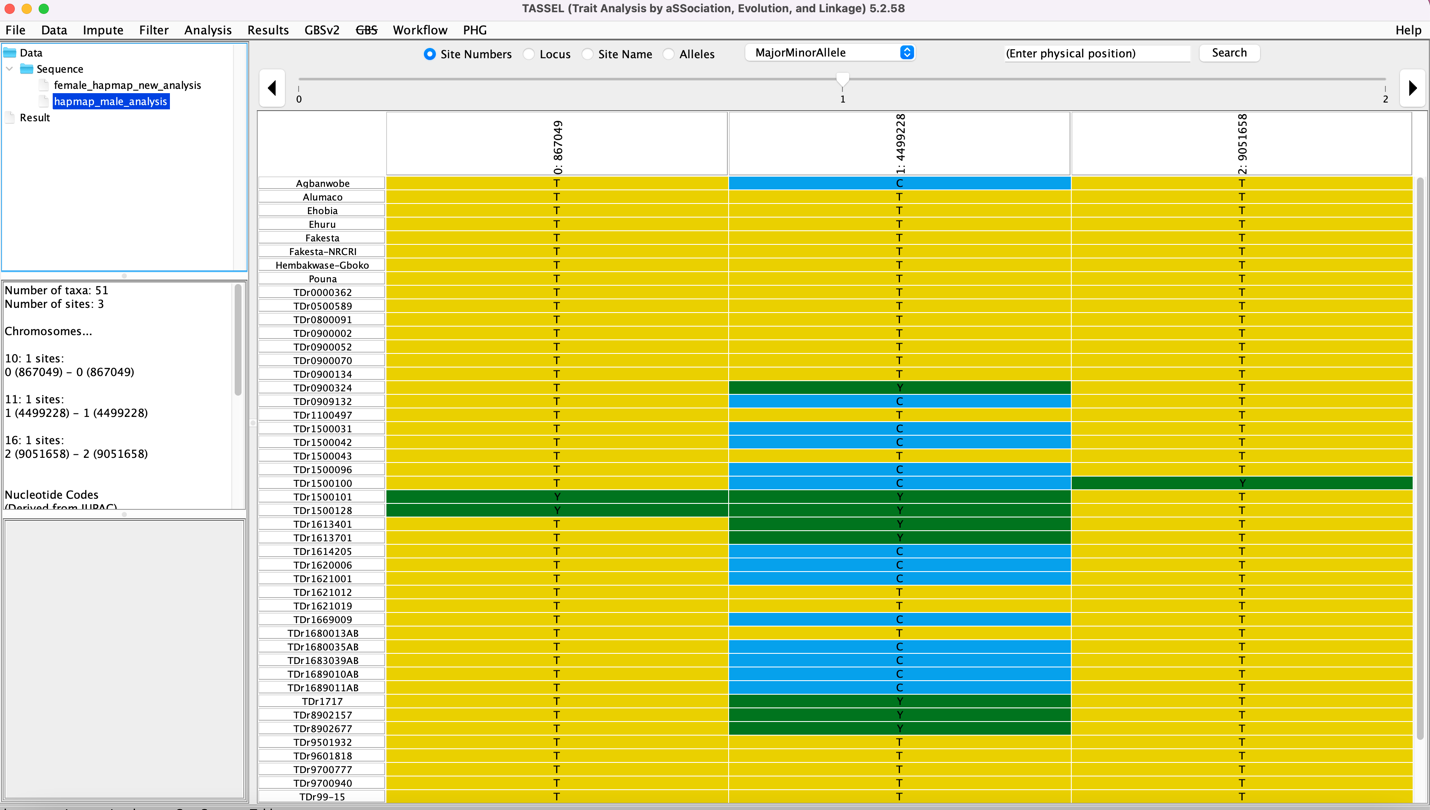


**Fig. S5.** Haplotype view of markers associated with plant sex in male plant of *D. rotundata* (green are heterozygote alleles while yellow and blue are for homozygote).
